# Solitary Metastasis of Gastric Cancer to Fibula: A Case Report

**DOI:** 10.5812/iranjradiol.3564

**Published:** 2012-09-17

**Authors:** Sepideh Hekmat, Tahereh Ghaedian, Hossein Barati, Mansour Movahed

**Affiliations:** 1 Department of Nuclear Medicine, Hasheminejad Hospital, Tehran University of Medical Sciences, Tehran, Iran

**Keywords:** Gastric Cancer, Neoplasm Metastasis, Fibula

## Abstract

Gastric cancer is one of the most common and most fatal neoplasms in human. Its skeletal metastasis is less frequent, particularly when solitary. The objective of this article is to represent a case of solitary fibular metastasis from this cancer not reported before based on Medline search.

## 1. Introduction

Gastric cancer is among the first five cancers in the world regarding incidence and mortality rate (1, 2). The reported recurrence rate for early gastric cancer ranges between 1.4% and 3.4% (3). It has already been stated that osseous metastases from primary gastric neoplasms are uncommon and it is probable that solitary osseous metastasis from this source is quite rare (4). Among the reported cases of bone metastases, long bones are less likely to be involved (4). We hereby describe a case of gastric adenocarcinoma who returned with a solitary bone metastasis to the fibula as the first sign of recurrence about 6 months after diagnosis. We did not find any previously reported cases of the fibula as the sole site of recurrence in this cancer and it is also rarely found in other cancers.

## 2. Case Presentation

**Figure 1:**
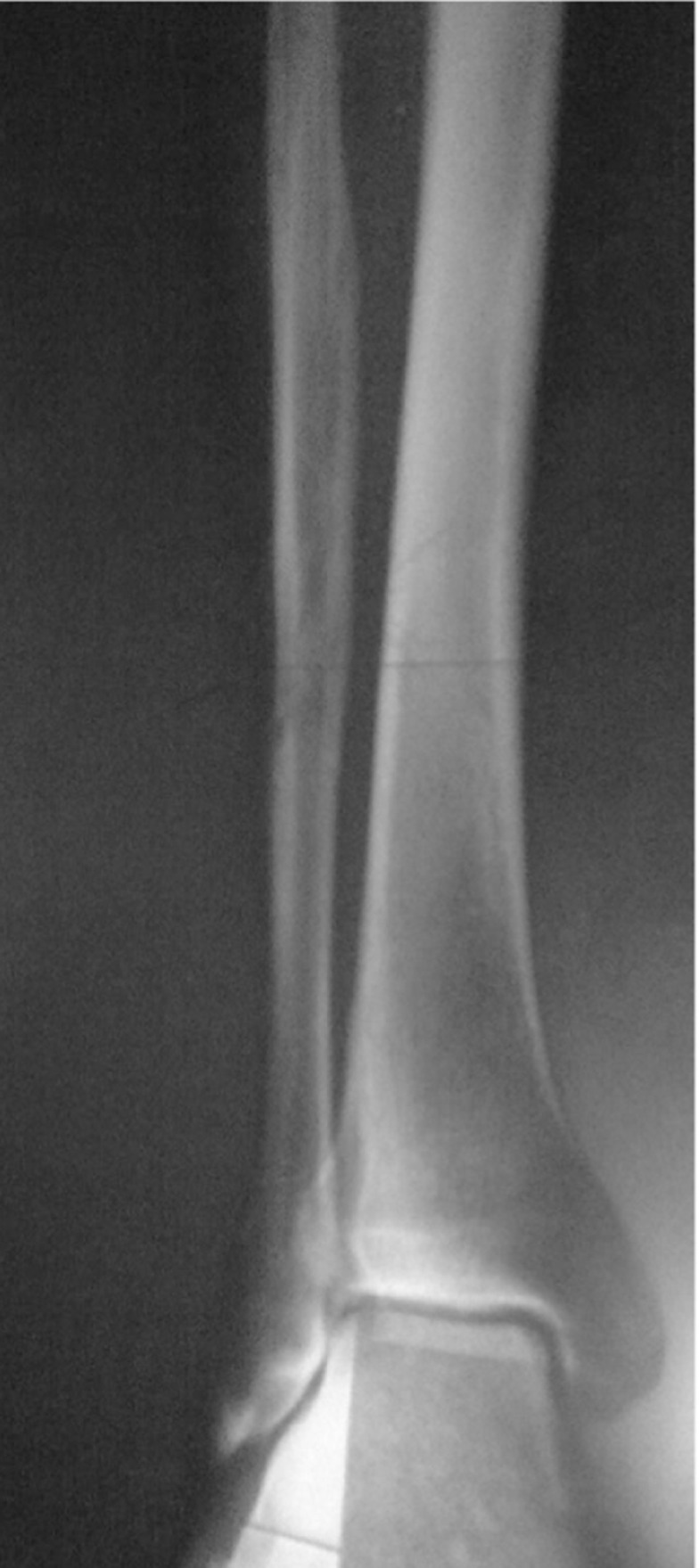
X-ray of the right tibia and fibula

**Figure 2:**
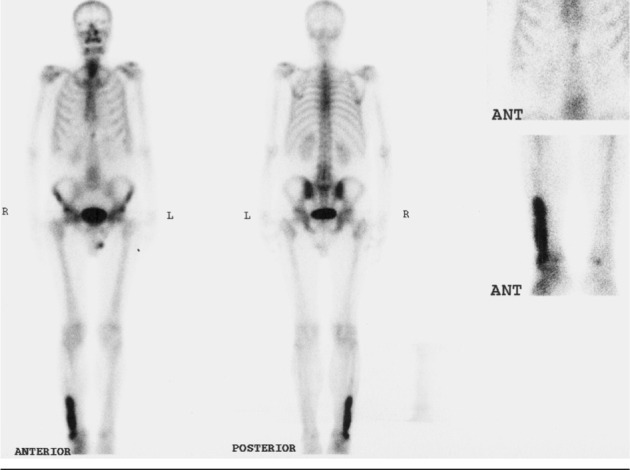
Whole body bone scan

**Figure 3:**
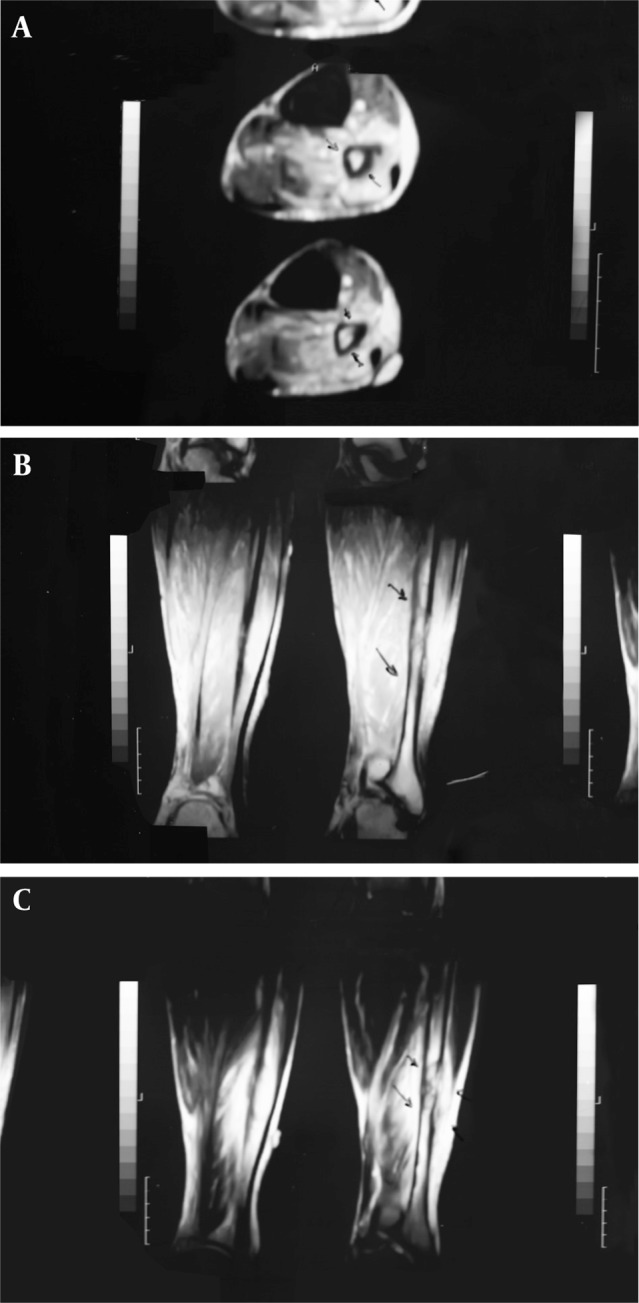
MRI of the right tibia A. Axial view, B. Sagittal view, C. Coronal view

The patient was a 65-year-old man who was referred to the nuclear medicine department with progressive pain and swelling in the distal portion of the right leg lasting for about 3 months. He denied any history of fever, trauma or prior pathology in this region. He was a known case of gastric adenocarcinoma for about 6 months before this presentation with a history of partial gastrectomy (resection of the proximal two thirds of the stomach) followed by chemotherapy (6 sessions for 6 months) and radiotherapy (40 sessions) without endoscopic evidence of residual tumor in the postsurgical gastric remnant. Early at presentation of right leg pain, the patient received a course of analgesics (NSAIDs) and then antibiotics without good response. With progression of the symptoms, further evaluation was carried out. Radiography of the right leg showed evidence of bone destruction and periosteal reaction in the distal diaphysis of the fibula. The tibia was normal (Figure 1). With suspicion of metastasis versus infection, bone scintigraphy was requested. Bone scan with 99mTc-MDP showed an expansile lesion with intense radiotracer uptake along the distal half of the right fibula (Figure 2). There was no other remarkable abnormality throughout the rest of the skeleton. These findings combined with the patient’s history and the destructive behavior of the lesion in radiography highlighted the possibility of a malignant process in this region. As further workup, MRI of the right leg was done which revealed abnormal signal intensity in the mid-diaphyseal portion of the right fibula involving medullary and cortical portions with soft tissue swelling and edema (Figure 3 A-C). So multiple CT-guided needle samples were obtained which finally confirmed metastatic adenocarcinoma on pathologic examination. After that, the patient underwent partial resection of the right fibula followed by radiotherapy (14 sessions) and chemotherapy (6 sessions for 6 months) .Other work ups for metastasis were negative.

## 3. Discussion

The incidence and mortality rate of gastric cancer are the fourth and second highest in the world, respectively as reported by WHO cancer statistics (1, 2). Recently, it has been reported that the most common form of recurrence in gastric cancer, including all stages, is peritoneal dissemination, followed by local recurrence, hepatic metastasis and distant metastasis in which bone metastasis is rare (3). However, in the study by Yoo et al., the most common recurrence pattern after peritoneal dissemination was hematogeneous recurrence (26.2%) followed by locoregional recurrence (19.3%) (5). They also demonstrated that these extra-abdominal patterns of recurrence occurred rarely without evidence of intra-abdominal metastasis (5). Bone metastasis in gastric cancer is a rare condition and varies from 0 to 17.5 % (4), although Kobayashi. et al. noted that this rate might be underestimated since bone scintigraphy is not performed as a routine clinical practice (6). The possible mechanisms of hematogeneous metastasis of gastric cancer are through: 1) the portal vein, 2) the venous system, other than the portal vein and 3) the lymphatic channels into the systemic circulation. Most cases of bone metastasis do not show liver involvement. Since most of the venous drainage from the stomach is via the portal vein and many cases of bone metastasis are associated with lymph node involvement, lymphatic channel drainage into the systemic circulation is the mechanism underlying bone metastasis (6). Bone metastases from gastric adenocarcinoma are usually osteolytic or less commonly mixed osteolytic-osteosclerotic (7). Osteosclerotic metastases are even rarer as noted in 2011 by Saito et al. presenting the seventh case of osteosclerotic bone metastasis from gastric cancer in the English-language literature (3). The spine was the most common metastatic site (66%), followed by the ribs (59%), pelvis (43%), femur (30%) and the skull (22%) (8). The least frequent metastatic sites were the shoulder girdle (17%), sacroiliac joint (7.2%), humerus (6.0%), sternum (4.2%) and the tibia (3.0%) (8), while to our knowledge, there is no previous report of the fibula as a site of bone metastasis in gastric adenocarcinoma. The predominance of metastatic disease in flat bones compared to the long bones is likely to be due to the presence of red marrow (4), supporting this fact that fibula as a long bone would be a rare site of metastasis as is the case in other cancers such as vaginal cancer, carcinoma of uterine cervix, lung cancer and colorectal cancer (9-13). On the other hand, as mentioned above, solitary bone metastasis as the sole sign of recurrence after hopefully curative resection without the disease spreading to other organs is much rarer (4). There is limited value for radiographic evaluation of bone metastases as symptoms caused by bone metastases frequently occur before being detectable by radiography whereas bone scintigraphy is generally accepted as the initial method of choice for bone metastasis assessment (6). Bone scintigraphy will show the extent of osseous lesions and it is also useful for demonstrating singularity versus multiplicity of the lesions and to identify asymptomatic lesions. Isotope scans often reveal more extensive lesions than those suggested by initial radiographs (13).

Usually, radiotherapy is sufficient in most cases of bone metastasis distal to the knee, but pain can be unremitting and resistant to radiotherapy. Options for treatment include local curettage with either bone grafting or cementation or below knee amputation (13). In some reported cases, surgical resection of the solitary bone lesion was accompanied with an acceptable survival (10, 14, 15). Our patient underwent surgical resection of the lesion together with radiotherapy and chemotherapy. The current patient is an unusual case of gastric adenocarcinoma with solitary bone metastasis to the fibula as the first and sole sign of recurrence. In spite of rarity, solitary bone metastasis to leg bones should be considered in the differential diagnoses in patients presenting with new onset of leg pain and a history of distant primary tumor such as gastric adenocarcinoma and it might be better to exclude this possibility first to avoid serious consequences of early misdiagnosis.
